# Results of a multicenter phase I/II trial of TCRαβ and CD19-depleted haploidentical hematopoietic stem cell transplantation for adult and pediatric patients

**DOI:** 10.1038/s41409-021-01551-z

**Published:** 2021-12-24

**Authors:** Wolfgang A. Bethge, Matthias Eyrich, Stephan Mielke, Roland Meisel, Dietger Niederwieser, Paul G. Schlegel, Ansgar Schulz, Johann Greil, Donald Bunjes, Arne Brecht, Jurgen Kuball, Michael Schumm, Vladan Vucinic, Markus Wiesneth, Halvard Bonig, Kasper Westinga, Stefanie Biedermann, Silke Holtkamp, Sandra Karitzky, Michaela Malchow, Christiane Siewert, Rupert Handgretinger, Peter Lang

**Affiliations:** 1 Department of Hematology, Oncology, Clinical Immunology and Rheumatology, Center for Internal Medicine, University Hospital Tuebingen, Tuebingen, Germany; 2 University Children’s Hospital Wuerzburg, Wuerzburg, Germany; 3 Karolinska Institutet and University Hospital, Department of Laboratory Medicine and Department of Medicine Huddinge, CAST, Karolinska Comprehensive Cancer Center, Stockholm, Sweden; 4 Department of Internal Medicine II, Wuerzburg University Medical Center, Wuerzburg, Germany; 5 Division of Pediatric Stem Cell Therapy, Department of Pediatric Oncology, Hematology and Clinical Immunology, Medical Faculty, Heinrich-Heine-University, Duesseldorf, Germany; 6 University Leipzig, Leipzig, Germany; 7 Department of Pediatrics, University Medical Center Ulm, Ulm, Germany; 8 University Children’s Hospital Heidelberg, Heidelberg, Germany; 9 Department of Internal Medicine III, University of Ulm, Ulm, Germany; 10 KMT-Abteilung, Helios Dr. Horst Schmidt Kliniken, Wiesbaden, Germany; 11 Department of Haematology and Center for Translational Immunology, University Medical Centre, Utrecht, The Netherlands; 12 CureVac RE GmbH, Tuebingen, Germany; 13 Department of Hematology/Oncology, University Children’s Hospital Tuebingen, Tuebingen, Germany; 14 Institute of Transfusion Medicine, University of Ulm, Ulm, Germany; 15 Institute for Transfusion Medicine and Immunohematology, and German Red Cross Blood Service BaWüHe, Goethe University, Frankfurt, Germany; 16 Cell Therapy Facility, University Medical Center Utrecht, Utrecht, The Netherlands; 17 Miltenyi Biotec, Bergisch Gladbach, Germany; 18 Miltenyi Biomedicine, Bergisch Gladbach, Germany

**Keywords:** Bone marrow transplantation, Haematological diseases

## Abstract

Hematopoietic stem cell transplantation (HSCT) from haploidentical donors is a viable option for patients lacking HLA-matched donors. Here we report the results of a prospective multicenter phase I/II trial of transplantation of TCRαβ and CD19-depleted peripheral blood stem cells from haploidentical family donors after a reduced-intensity conditioning with fludarabine, thiotepa, and melphalan. Thirty pediatric and 30 adult patients with acute leukemia (*n* = 43), myelodysplastic or myeloproliferative syndrome (*n* = 6), multiple myeloma (*n* = 1), solid tumors (*n* = 6), and non-malignant disorders (*n* = 4) were enrolled. TCR αβ/CD19-depleted grafts prepared decentrally at six manufacturing sites contained a median of 12.1 × 10^6^ CD34^+^ cells/kg and 14.2 × 10^3^ TCRαβ^+^ T-cells/kg. None of the patients developed grade lll/IV acute graft-versus-host disease (GVHD) and only six patients (10%) had grade II acute GVHD. With a median follow-up of 733 days 36/60 patients are alive. The cumulative incidence of non-relapse mortality at day 100, 1 and 2 years after HSCT was 5%, 15%, and 17% for all patients, respectively. Estimated probabilities of overall and disease-free survival at 2 years were 63% and 50%, respectively. Based on these promising results in a high-risk patient cohort, haploidentical HSCT using TCRαβ/CD19-depleted grafts represents a viable treatment option.

## Introduction

Hematopoietic stem cell transplantation (HSCT) from a haploidentical donor has evolved as the gold standard in alternative donor transplantation, i.e., in cases where a suitable HLA-matched donor is not timely available. Transplant numbers using this modality are increasing worldwide [1]. Today, several T-replete and T-deplete approaches are available. The rise of posttransplant cyclophosphamide (PT-Cy) as a broadly applied T-replete strategy has certainly fostered the use and applicability of the haplo approach in general. However, ex vivo T-cell depletion (TCD) is still the method of choice for minimizing T-cell mediated alloreactivity without the need for prolonged immunosuppression and low rates of acute and chronic graft-versus-host disease (GVHD). Techniques for ex vivo TCD have undergone a constant evolution and have shifted from a pan TCD, e.g., CD34^+^ cell selection, to more selective T-cell depletion approaches [2–4].

Evidence emerged that T-cells endowed with the gamma/delta T-cell receptor (TCRγδ^+^ T-cells) harbor reduced potential for alloreactivity [5, 6]. Moreover, human allograft recipients with higher than normal TCRγδ^+^ T-cell reconstitution showed a significantly improved 5-year leukemia-free and overall survival (OS) without increased GVHD incidence [7], indicating that TCRγδ^+^ T-cells might contribute to immune control over leukemia and infectious agents in humans. These data led to development of a novel TCD approach (TCRαβ/CD19 depletion), which selectively depletes TCRαβ^+^ T and B cells from the graft but retains large numbers of TCRγδ^+^ T-cells in addition to all other accessory cells. Depletion of CD19^+^ B cells serves as prophylaxis of posttransplant lymphoproliferative disease. The excellent technical performance and clinical feasibility of this approach was already demonstrated [8, 9] in several monocenter trials or case reports [10–19]. However, a broader application of this novel technique required confirmation of these data in a prospective, multicenter setting with decentralized GMP-compliant graft manipulation. Here, we report our prospective, multicenter phase I/II trial with patients receiving TCRαβ/CD19-depleted haploidentical allografts after a reduced-intensity conditioning (RIC) [20].

## Patients and methods

### Patients

Sixty patients with various malignant or non-malignant diseases lacking an HLA-matched stem cell donor and with an urgent indication for HSCT were consecutively enrolled in this study in 12 participating centers between May 2013 and November 2016. The study protocol was approved by the German Federal Agency “Paul-Ehrlich-Institut”, the Dutch Ministry of Health, Welfare and Sport and the relevant ethics committees in Germany and the Netherlands. Written informed consent was obtained from either patients or their legal guardians and donors in accordance with the Declaration of Helsinki.

### Graft processing

The hematopoietic stem cell graft was prepared decentralized at six different manufacturing sites across two countries. The stem cell apheresis product was depleted of TCRαβ^+^ and CD19^+^ cells using the automated CliniMACS^®^ Plus device (Miltenyi Biotec, Bergisch Gladbach, Germany).

The graft was targeted to contain ≥4 × 10^6^ CD34^+^ progenitor cells/kg and ≤25 × 10^3^ TCRαβ^+^ T-cells/kg and 1 × 10^5^ CD20^+^ B cells/kg, respectively.

### Treatment protocol

The RIC consisted of fludarabine (1 × 40 mg/m^2^, days −8 to −5), thiotepa (2 × 5 mg/kg, day −4), melphalan (1 × 70 mg/m^2^, days −3 to −2) and Anti-thymocyte globulin [(ATG) Grafalon, Neovii] given on days −12 to −9 [15 mg/kg (*n* = 17) or 30 mg/kg (*n* = 36, after amendment)]. Patients with treatment-refractory hematologic malignancies not in complete remission (CR) and patients with an increased risk of graft failure could receive total nodal irradiation (7 Gy on day −1) according to hospital routine instead of ATG (*n* = 7). GVHD prophylaxis consisted of mycophenolate mofetil (2 × 20 mg/kg, day −1 until day 30 posttransplantation).

### Engraftment, chimerism, and graft-versus-host disease

Engraftment was defined as the first of three consecutive days on which the absolute neutrophil count (ANC) was ≥500/µl, platelet recovery as independence from platelet substitution for at least 7 days with a platelet count ≥20,000/µl. Primary graft failure was defined as the failure to achieve an ANC >500/µl at day +28 and secondary graft failure as initial neutrophil engraftment followed by a decline in ANC <500/µl that is unresponsive to growth factor therapy. Chimerism was assessed by PCR analysis of until 1 year after HSCT.

Acute and chronic GVHD were classified according to consensus criteria [21, 22].

### Survival

OS was defined as the probability of survival from the time of HSCT to time of death or of last follow-up. Non-relapse mortality (NRM) was defined as the probability of death from any cause other than recurrence. Disease-free survival (DFS) was defined as the probability of survival, without evidence of malignancy at any time after transplantation. GVHD and relapse-free survival (GRFS) was determined according to Holtan et al. [23].

Details on stem cell graft preparation, immune reconstitution, supportive care, and statistics are described in the Supplementary Information.

## Results

### Patient demographics

Table 1 shows the main patient and transplant characteristics. Sixty patients with a median age of 18.5 years (range 1–63) were enrolled.

**Table 1:** Patient characteristics and graft composition.

Patients	
Diagnosis (*n* = 60)	
AML	25
ALL	17
MDS/MPS	6
MM, relapsed or refractory	1
Acute undifferentiated leukemia	1
Non-malignant diseases	4
Solid tumors	6
Age^a^ (years)	18.5 (1–63)
Weight^a^ (kg)	54.1 (10.8–143)
Disease status prior to haplo HSCT	
Pat. with malignancy and 1^st^ HSCT	36
CR1	12
>CR1	13
PR	1
SD	2
Non-remission	8
Pat. with malignancy and subsequent HSCT	20
>CR1	13
PR	1
Non-remission	6
Graft composition^a^ (cells/kg)	
Stem cells (CD34^+^)	12.1 × 10^6^ (4.0–54.9)
TCRαβ^+^ cells	14.2 × 10^3^ (2.2–64.3)
TCRγδ^+^ cells	10.4 × 10^6^ (1.1–45.0)
B cells (CD20^+^)	3.8 × 10^4^ (0.2–72.7)
NK cells (CD56^+^)	45.3 × 10^6^ (6.8–182.2)

*AML* acute myeloid leukemia, *ALL* acute lymphoblastic leukemia, *CR* complete remission, *HSCT* hematopoietic stem cell transplantation, *MDS/MPS* myelodysplastic or myeloproliferative syndrome, *MM* multiple myeloma, *PR* partial remission, *SD* stable disease; disease status: degree of remission achieved after chemotherapy prior to study HSCT (haplo HSCT), *Pat. with 1st HSCT* these patients received their 1^st^ HSCT within this study, *Pat. with subsequent HSCT* these patients had relapsed after previous HSCTs and received a 2^nd^ or 3^rd^ HSCT within this study.

^a^Median values.

Twenty-five patients suffered from acute myeloid leukemia [(AML), *n* = 16 with relapsed or primary refractory, *n* = 9 with high-risk AML in CR1], 17 patients had acute lymphoblastic leukemia [(ALL), *n* = 13 with relapsed or primary refractory ALL, *n* = 4 with high-risk ALL in CR1)] and six patients had refractory myelodysplastic or myeloproliferative syndrome (MDS/MPS). Further hematologic malignancies included acute undifferentiated leukemia (*n* = 1) and multiple myeloma (*n* = 1). Six pediatric patients suffered from solid tumors (relapsed metastatic soft tissue sarcoma, *n* = 4; alveolar rhabdomyosarcoma, *n* = 1; relapsed metastatic nmyc-negative neuroblastoma, *n* = 1) and four patients had non-malignant diseases (severe combined immune deficiency, *n* = 1; Wiskott–Aldrich Syndrome, *n* = 1; lysosomal storage disorder, *n* = 1; sickle cell anemia, *n* = 1). Of the 56 patients with malignancies, 38 were transplanted in CR (*n* = 12 CR1, *n* = 26 > CR1). Two and 14 patients were transplanted in partial (PR) and non-remission (minimal residual disease burden >10^4^ or >5% blasts or transplanted in aplasia), respectively. Two patients with solid tumors (radiologically visible residual tumor) were transplanted in stable disease. Twenty patients with malignancies had received one or two previous HSCTs.

Donors of G-CSF mobilized peripheral blood stem cells were ≥2 HLA loci (2–3 loci *n* = 9, 4–5 loci *n* = 51) mismatched parents or siblings. The specific demographics for the pediatric and adult cohort are shown in Supplementary Table 1.

### Graft processing

**Fig. 1:**
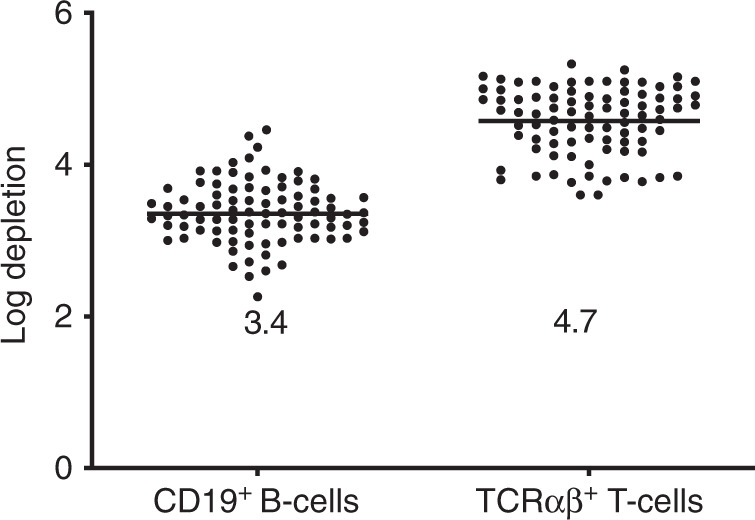
Performance of the TCRαβ- and CD19 depletion. Median logarithmic depletion of TCRαβ^+^T- and CD19^+^ B cells in 87 depletion procedures with the CliniMACS Plus device.

The median log depletion of TCRαβ^+^ T-cells and CD19^+^ B cells was 4.7 (range 3.6–5.3) and 3.4 (range 2.3–4.5), respectively (Fig. 1). The target dose of 4 × 10^6^ CD34^+^ progenitor cells/kg (Table 1) was achieved for all patients: a median of 12.1 × 10^6^ (range 4.0–54.9) CD34^+^ cells/kg and 14.2 × 10^3^ (range 2.2–64.3) TCRαβ^+^ T-cells/kg were infused. The graft contained high numbers of NK and TCRγδ^+^ T-cells: all patients received a median of 10.4 (range 1.1–45) × 10^6^ TCRγδ^+^ T-cells/kg and 45.3 (range 6.8–182.2) × 10^6^ NK-cells/kg. All but two patients received a fresh graft. One adult and one pediatric patient received a cryopreserved graft.

### Engraftment

Median time to neutrophil and platelet engraftment was 13 (range 9–41) and 15 (range 11–38) days, respectively.

Graft failure occurred in nine patients, six children and three adults. Indications included non-malignant diseases (*n* = 3), rhabdomyosarcoma (*n* = 1), refractory MDS/MPS (*n* = 2) and acute leukemia (*n* = 3). Initially, ATG dose as per protocol was set to 15 mg/kg with the intention of improving immune recovery. However, after occurrence of four cases of graft failure, the protocol was amended and the total dose of ATG was increased to 30 mg/kg. After the amendment, the graft failure rate decreased to 12.2% for all and to 7.3% for patients with hematologic malignancies. All patients with graft failure except one child, who died from sepsis could be rescued after re-conditioning and a secondary HSCT (*n* = 7) or by infusion of an autologous back-up (*n* = 1). Final engraftment was achieved in 59/60 patients (Table 2, details on pediatric and adult patients are shown in Supplementary Table 2).

**Table 2:** Engraftment, GVHD and survival.

Engraftment	*n*	
Median time to ANC ≥ 500/µl (days)	13	(9–41)
Median time to PLT ≥ 20,000/µl (days)	15	(11–38)
Initial engraftment	51	85%
Secondary engraftment		
(after re-conditioning and 2nd HSCT)^a^	8	13%
early mortality	1	2%
Final engraftment	59	98%

GvHD	*n*	%
**Acute**^b^		
Grade 0	31	52
1	23	38
2	6	10
3–4	0	0
Organ involvement		
Skin	26	43
Skin + gut	3	5
**Chronic**^c^		
Mild	5	10
Moderate	6	13
Severe	4	8
Organ involvement		
Skin	12	26
Lung	1	2
Mouth, eyes, GI tract, muscles, fascia, joints, genitalia	1	2
GI tract, gut, liver	1	2

Causes of death^d^	*n*	%	days post HSCT
Relapse	12	20	287 (68–809)^e^
ARDS	2	3	61,196
Bronchiolitis obliterans	1	2	188
Multi organ failure	1	2	18
Demyelinating neuropathy	1	2	131
Cardiac arrest	1	2	167
Adenovirus infection	3	5	123,259,505
Sepsis	1	2	33

*ANC* absolute neutrophil count, *ARDS* acute respiratory distress syndrome, *GI* gastro-intestinal, *GVHD* graft-versus-host disease, *HSCT* hematopoietic stem cell transplantation, *n* number of patients, *NRM* non-relapse mortality, *PLT* platelet count.

^a^One patient with sickle cell disease received an autologous stem cell back-up product.

^b^The maximum grading of acute GVHD per patient is reflected in this analysis.

^c^Patients were considered evaluable for chronic GvHD if they engrafted and survived for 100 days (*n* = 48). One adult patient, who developed chronic GVHD after infusion of a donor lymphocyte infusion (DLI, not planned per protocol, however, a total of ten patients of the study population received DLI) was excluded from this analysis.

^d^For one patient cause of death was not reported.

^e^Median values.

On day 28, 41 out of 45 evaluable patients had complete chimerism and another four patients had mixed chimerism. On day 100, peripheral T-cell chimerism was completely donor-type in 44 of 47 (94%) evaluable patients and mixed in three patients.

### Graft-versus-host disease

In total, 31/60 patients had no symptoms of acute GVHD (aGVHD), and 23 patients experienced grade I aGVHD. Grade II aGVHD occurred in six patients (Table 2)—five adults and one child (Supplementary Table 2). None of the patients developed grade III–IV aGVHD. Affected organs were skin only, except for one adult and two pediatric patients with gut involvement. The cumulative incidence of aGVHD ≥grade II was 10.1%, respectively.

Patients who had engrafted and were alive 100 days after transplantation were evaluable for chronic GVHD (cGVHD). Of 48 evaluable patients, five, six, and four patients experienced mild, moderate and severe cGVHD, respectively (Table 2). The cumulative incidence of total and moderate/severe cGVHD was 31.3% and 20.9%, respectively.

### Infections

**Fig. 2:**
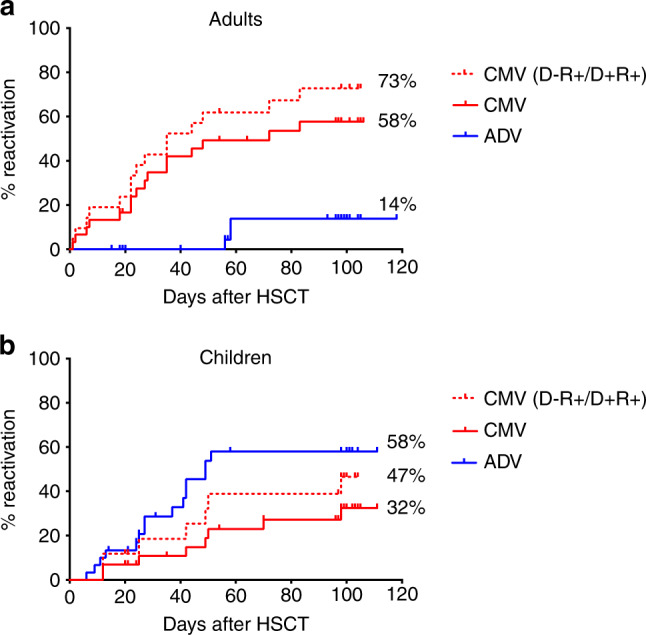
Cumulative incidence of virus reactivation. Patients were screened for adenovirus (ADV) DNA (stool, urine, and blood) and for cytomegalovirus (CMV) DNA (blood, stool, and throat); cumulative incidences of positive findings are shown for the adult (**a**) and pediatric (**b**) patient cohort, respectively, and for donor/recipient pairs with CMV IgG seropositive recipient (D−R+/D+R+); no events occurred in seronegative recipients (D−R−/D+R−).

Cytomegalovirus (CMV) reactivation was more frequent among adult compared to pediatric patients whereas more children than adults reactivated adenovirus [(ADV) Fig. 2]. The cumulative incidence for CMV reactivation in the first 100 days after transplantation was 46% for the whole patient cohort. In adult and pediatric patients CMV reactivation was 58% and 32%, in CMV positive recipients 73% and 47%, respectively. Two adults developed a CMV-associated colitis and hepatitis; none of the children developed CMV disease.

The cumulative incidence of ADV-specific DNAemia was 36% for all patients and 14% and 58% in adult versus pediatric patients, respectively. Two adults and nine children developed ADV-associated disease.

Two patients reactivated Epstein-Barr virus. However, there were no cases of posttransplant lymphoproliferative disorder. Details on infection in the adult and pediatric cohort are shown in Supplementary Table 3.

**Fig. 3:**
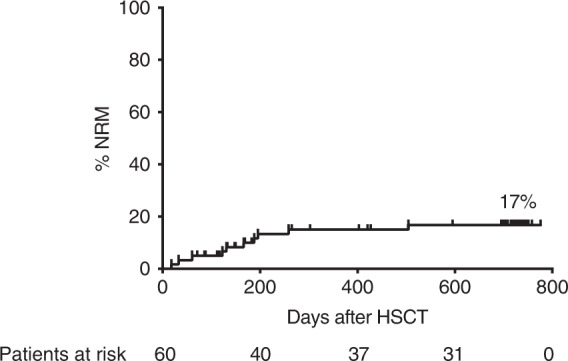
Non-relapse mortality. Cumulative incidence of non-relapse mortality (NRM) until 2 years posttransplant.

Except for one patient who died from acute respiratory distress syndrome due to metapneumovirus infection no other infection-related deaths were observed during the first 100 days. However, with a longer follow up, three children died due to ADV infection. The cumulative incidence of NRM was 5%, 15%, and 17% for all patients at day 100, 1 and 2 years after transplantation, respectively (Fig. 3, NRM for adults and children is shown in Supplementary Fig. 1).

### Immune reconstitution

CD3^+^ T-cell recovery started early with a median of 28 days required to reach a CD3^+^ cell count >100 cells/µl. Both CD3^+^ CD4^+^ T-cell and CD3^+^CD8^+^ T-cell counts >100 cells/µl were reached after a median of 183 days. CD3^+^, CD3^+^CD4^+^, and CD3^+^CD8^+^ T-cell populations reached normal levels at month 12.

**Fig. 4:**
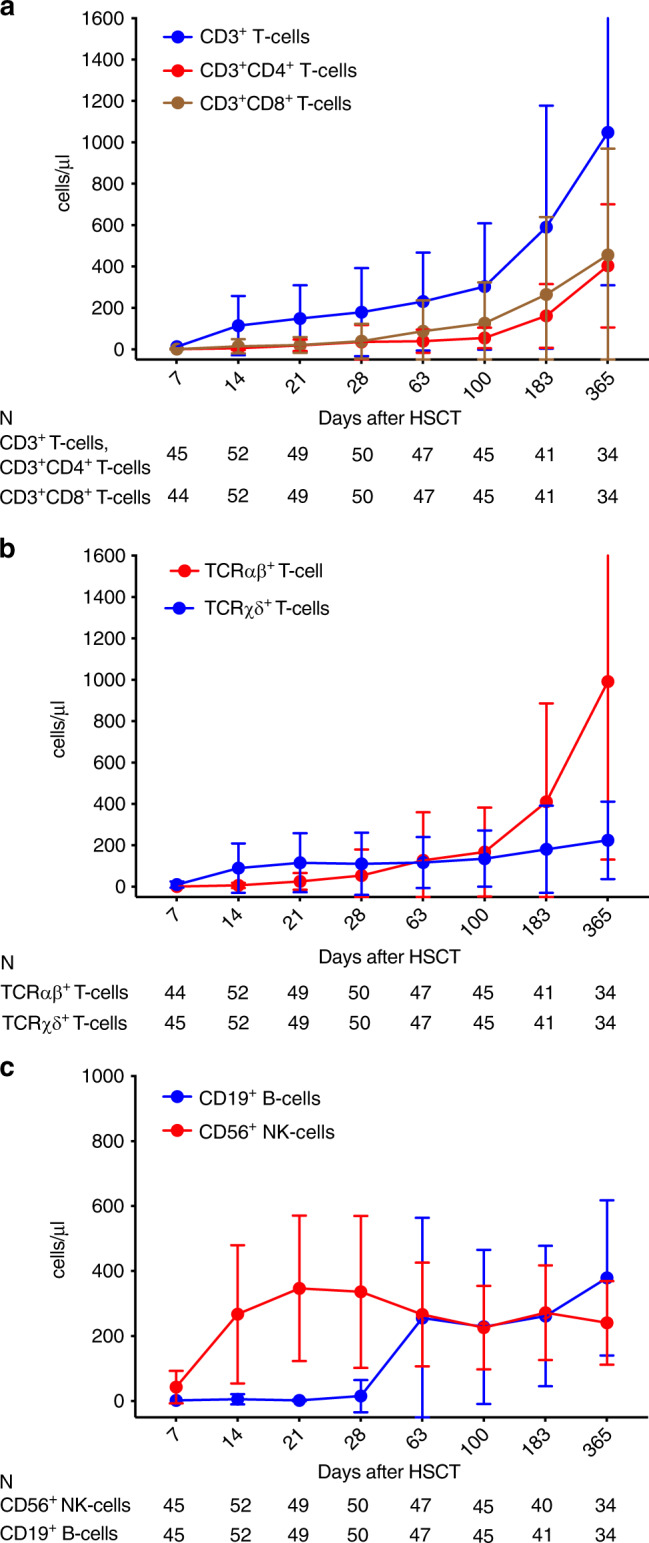
Immune reconstitution of different lymphocyte subsets after HSCT. Reconstitution of CD3^+^ T-cells, CD3^+^CD4^+^ T-cells, and CD3^+^CD8^+^ T-cells (**a**), TCRαβ^+^ and TCRγδ^+^ T-cells (**b**) and CD19^+^ B- and CD56^+^ NK-cells (**c**) after transplantation of TCRαβ/CD19-depleted allografts. Points represent the mean values ± standard deviations at each time point.

During the first 4 weeks after transplantation the TCRγδ^+^ T-cells were the predominant subset; after this time the TCRαβ^+^ T-cell subset started to increase. Recovery of CD56^+^ NK-cells was rapid: 14 days after transplantation a mean of 267 NK-cells/µl (range 1–999) was reached. Figure 4 shows the immune reconstitution for the various lymphocyte subsets during the first year after transplantation. The TCRγδ^+^ repertoire undergoes significant clonal evolution in the early posttransplantion period (Supplementary Figs. 2 and 3). Patterns of immune reconstitution were very similar between pediatric and adult patients (Supplementary Fig. 4).

### Relapse

**Fig. 5:**
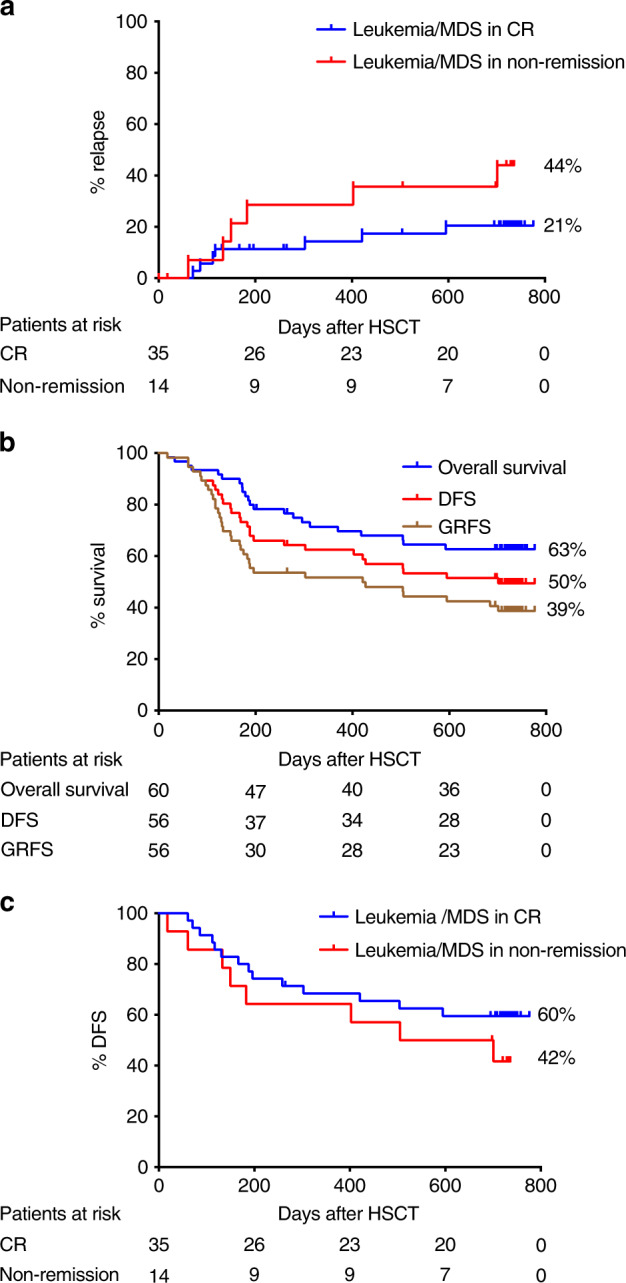
Relapse rate, overall survival, disease-free survival, and GVHD-relapse-free survival for the whole patient cohort. **a** Relapse rates in patients with leukemia/myelodysplastic syndrome (MDS) transplanted in complete remission (CR) versus patients with leukemia/MDS transplanted in non-remission. **b** Disease-free survival (DFS), overall survival (OS), and GVHD-, relapse-free survival (GRFS) of the whole patient group. **c** DFS of patients with leukemia/MDS transplanted in CR versus patients with leukemia/MDS transplanted in non-remission.

**Fig. 6:**
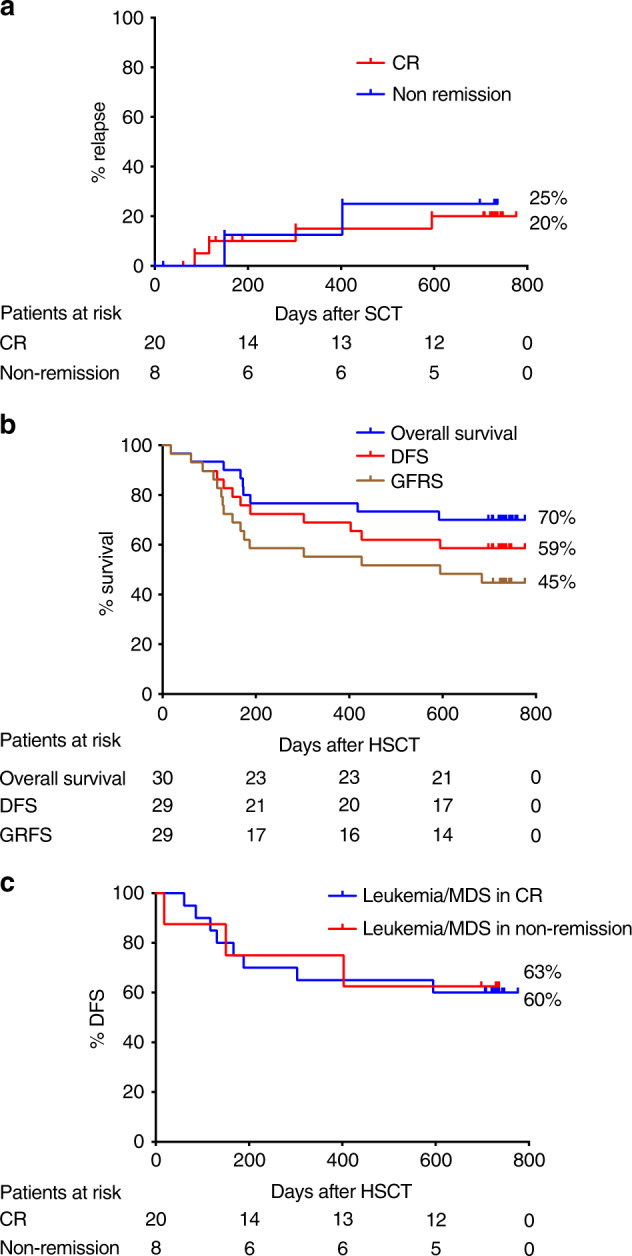
Relapse rate, overall survival, disease-free survival, and GVHD-relapse-free survival for adult patients. **a** Relapse rates in adult patients with leukemia/myelodysplastic syndrome (MDS) transplanted in complete remission (CR) versus patients with leukemia/MDS transplanted in non-remission. **b** Disease-free survival (DFS), overall survival (OS), and GVHD-, relapse-free survival (GRFS) of the adult cohort. **c** DFS of adult patients with leukemia/MDS transplanted in CR versus adult patients with leukemia/MDS transplanted in non-remission.

Eighteen patients relapsed and 12 of them died due to progressive disease. The cumulative incidence of relapse at 2 years for patients with malignancies was 33%. When evaluating only those patients with leukemia or MDS transplanted in CR the cumulative incidence of relapse was 21% (Fig. 5a). Among adults, the incidence of relapse was similar between those patients with leukemia or MDS transplanted in CR (20%) or in non-remission (25%); whereas for the pediatric cohort, the incidence of relapse was clearly lower when the patients were transplanted in CR, 21% compared to 67% in case the patients were transplanted in non-remission (Fig. 6a and Supplementary Fig. 5a).

### Survival

With a median follow-up of 733 days (range 265–776 days) 36/60 patients are alive. Leading cause of death was relapse (*n* = 12), followed by lethal ADV infection in three pediatric patients. Two patients died due to acute respiratory distress syndrome and one case each due to cardiac arrest, multi organ failure, demyelinating neuropathy, and bronchiolitis obliterans. One patient died due to sepsis after graft failure and for one patient cause of death was not reported (Table 2). One patient discontinued the study after withdrawal of informed consent.

The estimated probabilities of OS and DFS at 2 years were 63% and 50%, respectively (Fig. 5b). When analyzing the subgroup of patients who were transplanted for leukemia or MDS in CR versus patients with leukemia or MDS transplanted in non-remission DFS was 60% versus 42%, respectively (Fig. 5c).

In adults, the estimated probability of OS at 2 years was 70%. DFS for patients with leukemia or MDS was very similar, regardless if transplanted in CR (60%) or in non-remission (63%) (Fig. 6b, c). In children, OS and DFS at 2 years were 55 and 59% for patients with leukemia or MDS transplanted in CR (Supplementary Fig. 5b, c).

GRFS at 2 years was 39%, 45%, and 32% for the whole cohort and adult and pediatric patients, respectively (Figs. 5b and 6b, and Supplementary Fig. 5b.).

## Discussion

This study reports a multicenter prospective phase I/II clinical trial evaluating haploidentical HSCT after RIC for the treatment of adults and children with malignant and non-malignant diseases without a suitable HLA-identical donor. Haploidentical donors are increasingly used for allogeneic transplantation, fostered by the introduction of haploidentical HSCT after profound in vitro TCD by the group of Martelli et al. and later by the introduction of the PT-Cy strategy by Fuchs et al. [24, 25]. Ex vivo TCD is still the method of choice for minimizing T-cell mediated alloreactivity without the need for prolonged immunosuppression and yet low rates of acute and chronic GVHD. In addition, T-cell-depleted approaches might be the ideal platform for further applications of adoptive immunotherapy such as donor lymphocyte infusions [26], virus-specific T-cells, or lately allogeneic CAR T-cells. The haploidentical family donor is usually easily available for these adoptive approaches.

Recently, the immunomagnetic depletion of TCRαβ^+^ T- and CD19^+^ B cells cells has evolved to a fully automated and GMP-compliant operating procedure. Several single and multicenter retrospective studies already reported the safety and feasibility of TCRαβ/CD19 depletion as graft engineering approach [16, 18, 27].

Our data strongly support this by demonstrating that the depletion procedure is very reproducible and robust even in a multicenter setting. In this study, decentralized and cross-national production at six manufacturing sites was applied. Variability of TCRαβ/CD19-depleted grafts was low with a median of only 14 × 10^3^ and 12 × 10^6^ cells/kg for TCRαβ^+^ and CD34^+^ cells, respectively. Notably, there were no failures in almost 90 depletion procedures. Besides the RIC involving fludarabine/thiotepa/melphalan, the grafts highly enriched in innate effector cells such as TCRγδ^+^ T- and NK-cells most likely contributed to the low NRM rate, although our cohort contained partly older or heavily pretreated individuals including patients having received prior allogeneic or autologous HSCT.

Despite the RIC and the use of ATG early upfront of the stem cell infusion, we observed swift and stable engraftment in the majority of patients. Rate of graft failure after the amendment (ATG increased to 30 mg/kg) was 7.3% for patients with hematologic malignancies. All but one cases of graft failure were successfully rescued by secondary HSCT.

The observed low incidence of aGVHD of only 10% with no cases of grade III–IV aGVHD impressively highlights the strength of the ex vivo TCD approach. Of note, the majority of patients did not require any form of immunosuppression beyond day 30. These data seem promising in comparison to the reported GVHD incidence after PT-Cy and even after CD3/CD19 depletion [28].

In comparison to previous studies involving CD34 selection or CD3/CD19 depletion T-cell immune reconstitution was fast with a median time of 28 days to CD3^+^ cell count >100 cells/µl. The recovery for CD3^+^CD4^+^ and CD3^+^C8^+^ T-cells is delayed in the first 6 months, which is expected after TCD and reaches normal values after 1 year [29, 30]. Immune reconstitution, particularly of NK-, TCRγδ^+^ T- and Vδ2 cells in adult patients, seems promising if compared to recently reported data in PT-Cy-based HSCT [31–33].

The rapid reconstitution of TCRγδ^+^ T- and NK-cells in the early posttransplant period translated into a favorable incidence of infectious complications with only four infection-related deaths and a cumulative incidence of positive PCR of 36% for ADV and 46% for CMV. No cases of Epstein-Barr virus-related posttransplant lymphoproliferative disorder have been observed and even patients with active fungal infections at time of HSCT experienced no severe exacerbation posttransplant. CMV reactivation rate was as low as in the range otherwise reported for HLA-matched or PT-Cy-based HSCT [34, 35]. Albeit the overall low incidence of viral reactivations we observed four fatal cases of viral disease. We envisage that the adoptive transfer of CD45RA-depleted memory T-cells with a high frequency of antiviral T-cell clonotypes and a low GVHD-potential might be able to control such cases in the future.

The low rate of infectious complications and GVHD contribute to the low NRM of only 5% and 15% on day 100 and 1 year, respectively. This represents a large improvement in comparison to previous data with CD3/CD19 depletion (NRM 23% and 42% at day 100 and after 2 years, respectively) [28], and seems similar to protocols involving PT-Cy with NRM at day 100 of 11% [36].

OS and DFS at 2 years with 63% and 50%, respectively, seem encouraging in our study, given the high-risk patient population with most patients being in CR ≥1 or with persistent disease at time of HSCT. Cumulative incidence of relapse in CR patients was only 21% and in adults relapse rate in non-remission patients with only 25% almost approached the relapse rate in CR patients. Low rates of GVHD translate into a GRFS rate of 39% at 2 years, which is at least comparable to GRFS reported after HLA-matched related, unrelated or T-cell replete haploidentical HSCT [37, 38]. These results confirm or even excel previous reports on the use of haploidentical TCRαβ/CD19-depleted grafts [16, 18]. There is little published data on adult patients treated with a TCRαβ/CD19-depleted graft. OS and DFS of 70% and 59%, respectively, reported for the adult cohort of our study, and the DFS of 63% and the relapse rate of 25% in patients transplanted in non-remission seems encouraging.

In conclusion, this prospective clinical trial of haploidentical HSCT using TCRαβ/CD19 depletion and RIC demonstrates the feasibility of this approach in a multicenter setting. The clinical results show promise with high DFS, GRFS, and OS rates and low NRM even in a heavily pretreated patient population without the need for long-term immunosuppressive therapy. Especially the adult patient cohort seemed to benefit substantially from the early expanding, polyclonal TCRγδ^+^ T- and NK-cell pool. This strategy may now be evaluated in further larger clinical studies in comparison to other strategies for haploidentical HSCT, or even to fully HLA-matched transplantation.

## Supplementary information

Supplemental Information
