# Supplementary material for: Results of a multicenter phase I/II trial of TCRαβ and CD19-depleted haploidentical hematopoietic stem cell transplantation for adult and pediatric patients
Source: Bone Marrow Transplant. 2021 Dec 24;57(3):423–30. doi: 10.1038/s41409-021-01551-z (PMC8702395; doi:10.1038/s41409-021-01551-z)
Supplement: Supplementary file 1 — Supplemental Information [file 41409_2021_1551_MOESM1_ESM.pdf]

Supplementary information

Supplementary methods

Preparation of the hematopoietic stem cell graft

The stem cell apheresis product was depleted of $TCR\alpha\beta^+$ and $CD19^+$ cells using the automated CliniMACS® Plus device (Miltenyi Biotec, Germany) according to the manufacturer's instructions and institutional Standard Operating Procedures (SOPs). Briefly, up to 6×10^{10} leukocytes were incubated with one vial of TCR-αβ-Biotin reagent (biotinylated anti-TCRαβ antibody). After washing the cells were incubated with anti-Biotin- and CD19-coated magnetic microbeads. After an additional washing step, the cells were processed using the CliniMACS Depletion Tubing Set and the Depletion Program 3.1.

Immune reconstitution

The reconstitution of lymphocyte subsets was monitored on days 7, 14, 21, 28, 63, 100, month 6, and one year post-transplantation by flow cytometric analysis.

The reconstitution of T Vγ/δ repertoire was assessed by TCR Vγ/δ spectratyping on days 28, 63 and 100 post-transplantation.[1]

Supportive care

All patients received prophylaxis for viral, bacterial, and fungal infections according to institutional guidelines.

PCR analysis for fungal and viral reactivation was performed at baseline, once to twice weekly from day 4 to day 28, weekly until day 70 and at day 100. In case of suspected adenovirus (ADV) reactivation in stool or blood, administration of cidofovir (5 mg/kg) every 2 weeks and in case of increasing ADV copy numbers in blood, donation of ADV

specific donor T-cells was strongly recommended. Pre-emptive therapy for cytomegalovirus (CMV) infection with ganciclovir or foscarnet was recommended to start immediately after the first positive PCR. If CMV-DNA was detectable in urine or throat prior to transplant, eradication with ganciclovir or foscarnet should be tried before start of the conditioning regimen. In case of increasing copy numbers of Epstein Barr Virus in blood, pre-emptive administration of rituximab 375 mg/m^2 was recommended.

Statistical analysis

The probability of disease-free (DFS) and overall (OS) survival was assessed according to the Kaplan-Meier estimate. Engraftment, graft-versus-host disease (GVHD), non-relapse mortality (NRM), and relapse were calculated as cumulative incidence. NRM and relapse were considered to be reciprocal competing risks. Death was a competing risk for chronic GVHD and both death and graft failure were competing risks for acute GVHD. Competing risks were calculated using SAS software version 9.4 according to the cumulative incidence function.[2] Patients with non-malignant diseases were excluded from the analysis of DFS and relapse. Graphics were created and analysed using GraphPad Prism 8.0.

Supplementary results

Supplementary Table 1. Patient characteristics and graft composition

**Patients**	Adults (n=30)	Children (n=30)
**Diagnosis**		
ALL	7	10
AML	17	8
MDS/MPS	3	3
MM, relapsed or refractory	1	0
Acute undifferentiated leukemia	1	0
Solid tumors	0	6
Non-malignant diseases	1	3
**Age**[a] (years)	38.5 (20-63)	7 (1-17)
**Weight**[a] (kg)	75.8 (47-143)	27.7 (10.8-76.4)
**Disease status prior to haplo HSCT**		
Pat. with malignancy and 1st HSCT	21	15
CR 1	8	4
> CR 1	7	6
PR	0	1
SD	0	2
Non-remission	6	2
Pat. with malignancy and subsequent HSCT	8	12
> CR 1	5	8
PR	1	0
SD	0	0
Non-remission	2	4
**Graft composition** [a] (cells/kg)		
Stem cells ($CD34^+$)	$8.1x10^6$ (4.0 – 18.0)	$14.8x10^6$ (4.0 – 55.0)
$TCR\alpha\beta^+$ cells	$13.5 x10^3$ (2.2- 64.3)	$14.2x10^3$ (2.6- 40.6)
$TCR\gamma\delta^+$ cells	$8.9x10^6$ (1.1-30.7)	$16.2x10^6$ (1.3-45.0)
B cells ($CD20^+$)	$2.8x10^4$ (0.2-72.7)	$6.0x10^4$ (0.89-18.5)
NK cells ($CD56^+$)	$39.3x10^6$ (9.4-102.0)	$68.1x10^6$ (6.8-182.2)

CR complete remission, *HSCT* hematopoietic stem cell transplantation, *MDS/MPS* myelodysplastic/myeloproliferative syndrome, *MM* multiple myeloma, *n* number of patients, *PR* partial remission, *SD* stable disease, Disease status: degree of remission achieved after chemotherapy prior to study HSCT (haplo HSCT). Pat. with 1st HSCT: these patients received their 1st HSCT within this study. Pat. with subsequent HSCT: these patients had relapsed after previous HSCTs and received a 2nd or 3rd HSCT within this study. [a] Median (range)

Supplementary Table 2. Engraftment, GVHD, and survival

		Adults		Children	
**Engraftment**		n		n	
Median time to ANC ≥500/µl (days)		14	(9-41)	12	(10-18)
Median time to PLT ≥20,000/µl (days)		15	(12-38)	15	(11-27)
Initial engraftment		27	(90%)	24	(80%)
Secondary engraftment (after re-conditioning and 2nd SCT)		3[a]	(10%)	5	(17%)
TRM		0	(0%)	1	(3%)
Final engraftment		30	(100%)	29	(97%)
**GvHD**		n		n	
**Acute**[b]					
Grade	0	18	(60%)	13	(43%)
	1	7	(23%)	16	(53%)
	2	5	(17%)	1	(3%)
	3-4	0	(0%)	0	(0%)
Organ involvement					
	skin	11	(37%)	15	(50%)
	skin + gut	1	(3%)	2	(7%)
**Chronic**[c]					
	mild	3	(12%)	2	(9%)
	moderate	5	(20%)	1	(4%)
	severe	3	(12%)	1	(4%)
Organ involvement					
	skin	9	(36%)	3	(13%)
	lung	1	(4%)	0	(0%)
	Mouth, eyes, GI tract, muscles, fascia, joints, genitalia	1	(4%)	0	(0%)
	GI tract, gut, liver	0	(0%)	1	(4%)
**Causes of death**[d]		n		n	
Relapse		4	(13%)	8	(27%)
Acute respiratory stress syndrome		1	(3%)	1	(3%)
Bronchiolitis obliterans		1	(3%)	0	(0%)
Multi organ failure		1	(3%)	0	(0%)
Demyelinating neuropathy		1	(3%)	0	(0%)
Cardiac arrest		1	(3%)	0	(0%)
Adenovirus infection		0	(0%)	3	(10%)
Sepsis		0	(0%)	1	(3%)

ANC absolute neutrophil count, *GI* gastro-intestinal, *GVHD* graft-versus-host disease, *n* number of patients, *PLT* platelet count, [a] One patient with sickle cell disease received an autologous stem cell back-up product. [b] The maximum grading of aGVHD per patient is reflected in this analysis. [c] Patients were considered evaluable for chronic GVHD, if they engrafted and survived for 100 days (n=25 and 23 for the adult and pediatric cohort respectively). [d] Cause of death is unknown for one pediatric patient.

Supplementary Table 3. Infections associated with pathogens

Infections associated with pathogens	Adults (n)	Children (n)
Adenovirus infection	2	9
BK virus-associated hemorrhagic cystitis	3	2
Infection with clostridium difficile	0	2
Corona virus infection	1	1
Cytomegalovirus infection	2	0
Epstein-Barr virus infection	1	0
Fungal infections (pulmonary aspergillosis, fungal pneumonia)	4	2
Herpes infections (herpes zoster, herpes simplex virus and HHV6 infection)	3	4
Influenza virus infection	1	1
Listeria monocytogenes infection	1	0
Metapneumovirus infection	2	0
Norovirus infection	0	1
Parainfluenza virus infection	1	2
Pseudomonas infection	0	1
Rhinovirus infection	0	1
Rotavirus infection	1	1
Sapovirus infection	0	1
Sepsis	3	7
SIRS	0	1
Staphylococcus infection	1	1

HHV6 human herpesvirus 6, *n* number of patients, *SIRS* Systemic Inflammatory Response-Syndrome, infections with common pathogens are shown, individual patients could have experienced infections with several pathogens.

Supplementary Fig. 1 Non-relapse mortality. Cumulative incidence of NRM until 2 years posttransplant for the adult and pediatric patient cohort, respectively.

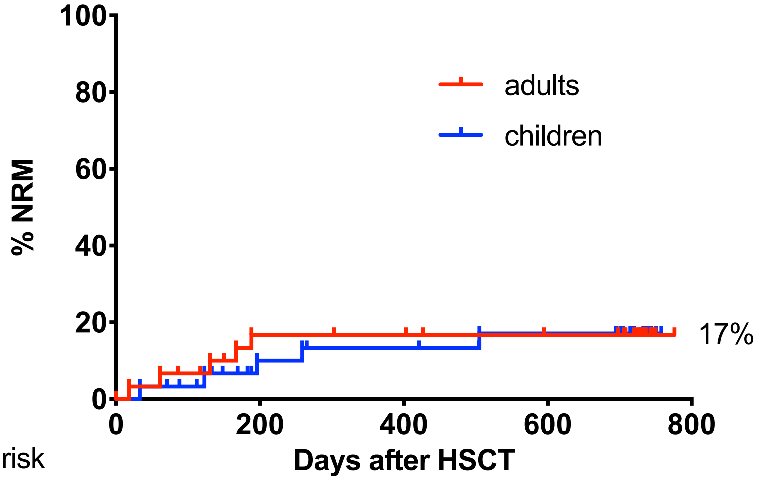

Patients at risk					
Adults	30	22	21	18	0
Children	30	18	16	13	0

Supplementary Fig. 2 Overall development of the Vγδ TCR repertoire at different time points after HSCT. The percentage of the overall Vγδ TCR repertoire of the donor and the recipient on day 28, day 63, and day 98 are shown.

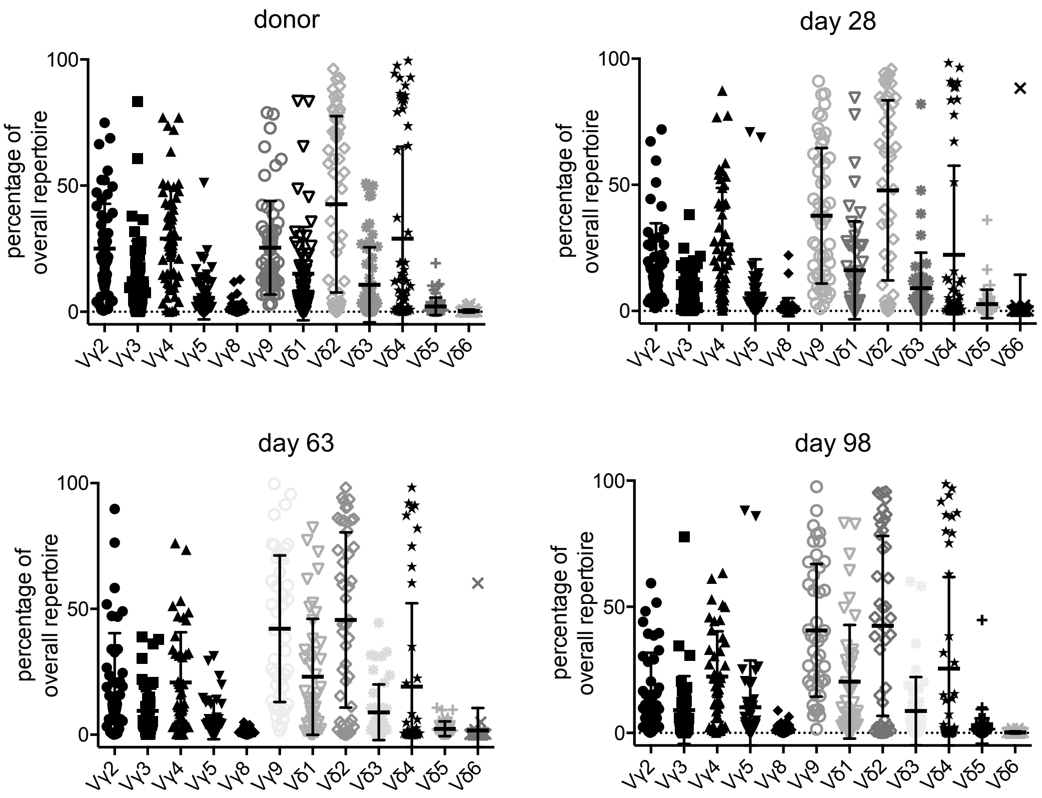

Supplementary Fig. 3 Representative individual development of the Vγδ TCR repertoire at different time points after hematopoietic stem cell transplantation. Representative individual development of the Vγδ TCR repertoire at different time points after hematopoietic stem cell transplantation**.** The Vγδ TCR repertoire of one individual patient in comparison to the donor repertoire.

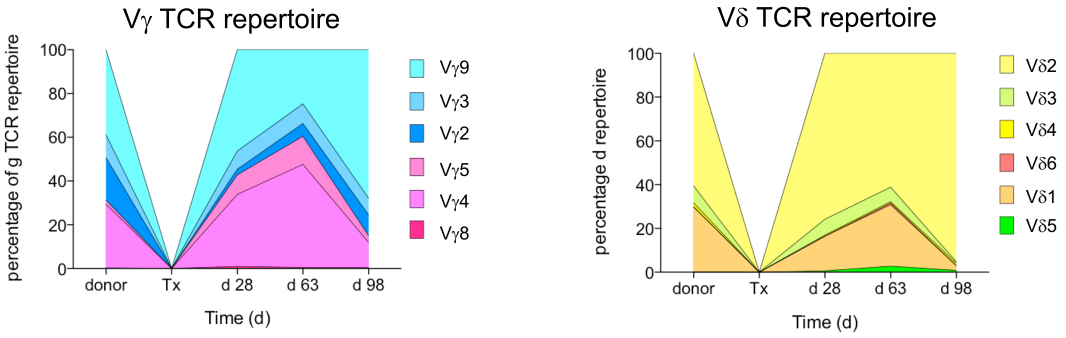

Supplementary Fig. 4 Immune reconstitution of different lymphocyte subsets after hematopoietic stem cell transplantation. Reconstitution of $CD3^+$ T-, $CD3^+CD8^+$ T- and $CD3^+CD4^+$ T-cells (**a**), TCRαβ$^+$- and TCRγδ$^+$- T-cells (**b**) and $CD19^+$ B- and $CD56^+$ NK-cells (**c**) after transplantation of TCRαβ/CD19 depleted allografts for the adult and pediatric cohort, respectively. Points represent the mean values ± standard deviations at each time point. Dashed lines represent the lower threshold of reference values for $CD3^+$ T cells (blue), $CD3^+CD4^+$ (red) and $CD3^+CD8^+$ T cells (brown).

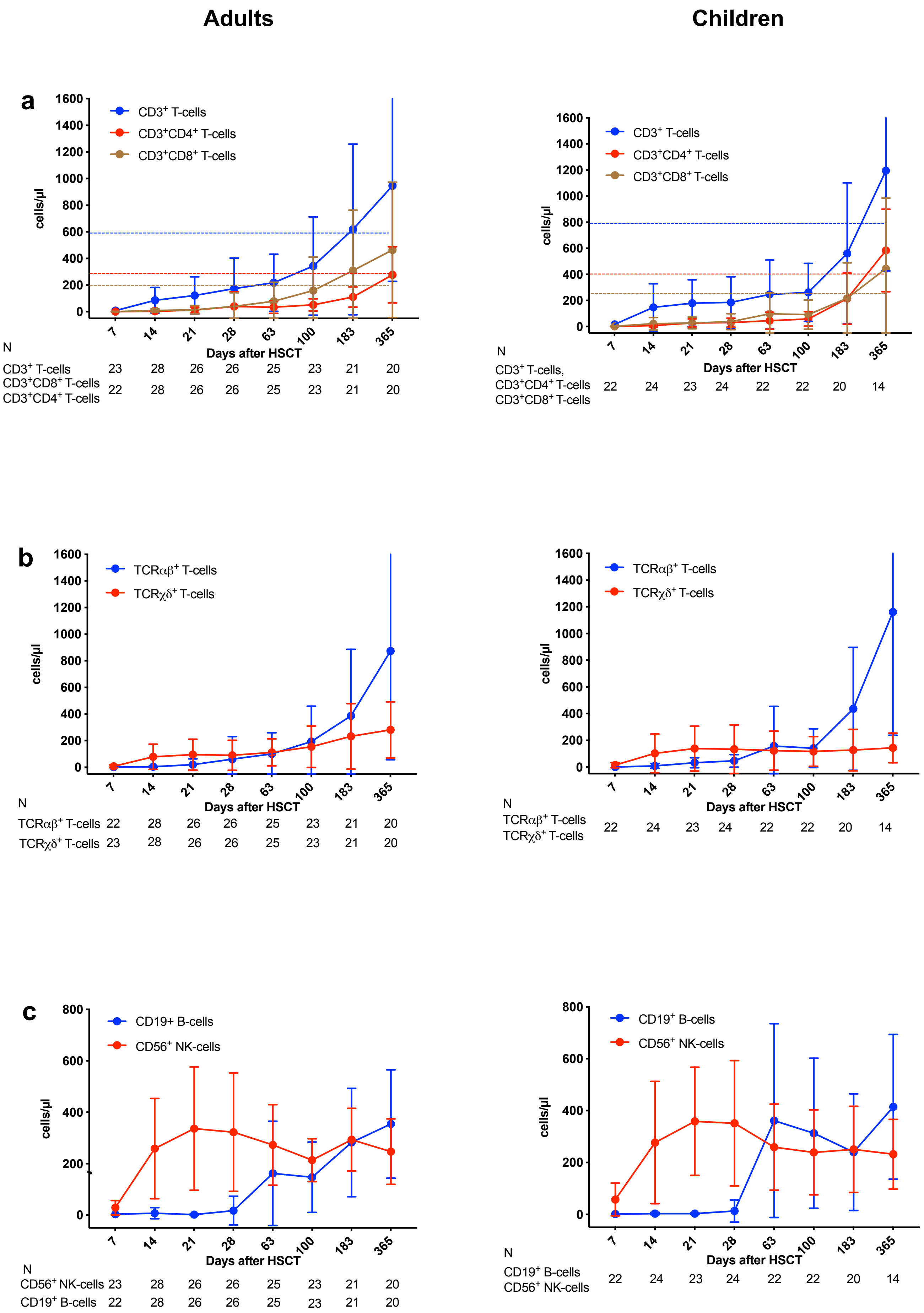
Adults
Children
a
CD3+ T-cells
CD3+CD4+ T-cells
CD3+CD8+ T-cells
cells/µl
Days after HSCT
N
CD3+ T-cells 23 28 26 26 25 23 21 20
CD3+CD8+ T-cells CD3+CD4+ T-cells 22 28 26 26 25 23 21 20
CD3+ T-cells, CD3+CD4+ T-cells CD3+CD8+ T-cells 22 24 23 24 22 22 20 14
b
TCRαβ+ T-cells
TCRχδ+ T-cells
TCRαβ+ T-cells 22 28 26 26 25 23 21 20
TCRχδ+ T-cells 23 28 26 26 25 23 21 20
TCRαβ+ T-cells TCRχδ+ T-cells 22 24 23 24 22 22 20 14
c
CD19+ B-cells
CD56+ NK-cells
CD56+ NK-cells 23 28 26 26 25 23 21 20
CD19+ B-cells 22 28 26 26 25 23 21 20
CD19+ B-cells CD56+ NK-cells 22 24 23 24 22 22 20 14

Supplementary Fig. 5. Relapse rate, overall survival, disease free survival, and GVHD-relapse-free survival for pediatric patients

a Relapse rates for pediatric patients with leukemia/myelodysplastic syndrome (MDS) transplanted in complete remission (CR) versus patients with leukemia/MDS transplanted in non-remission; **b** Disease-free survival (DFS), overall survival, and GVHD-relapse-free survival (GRFS); **c** DFS of pediatric patients with leukemia/MDS transplanted in CR versus patients with leukemia/MDS not in remission.

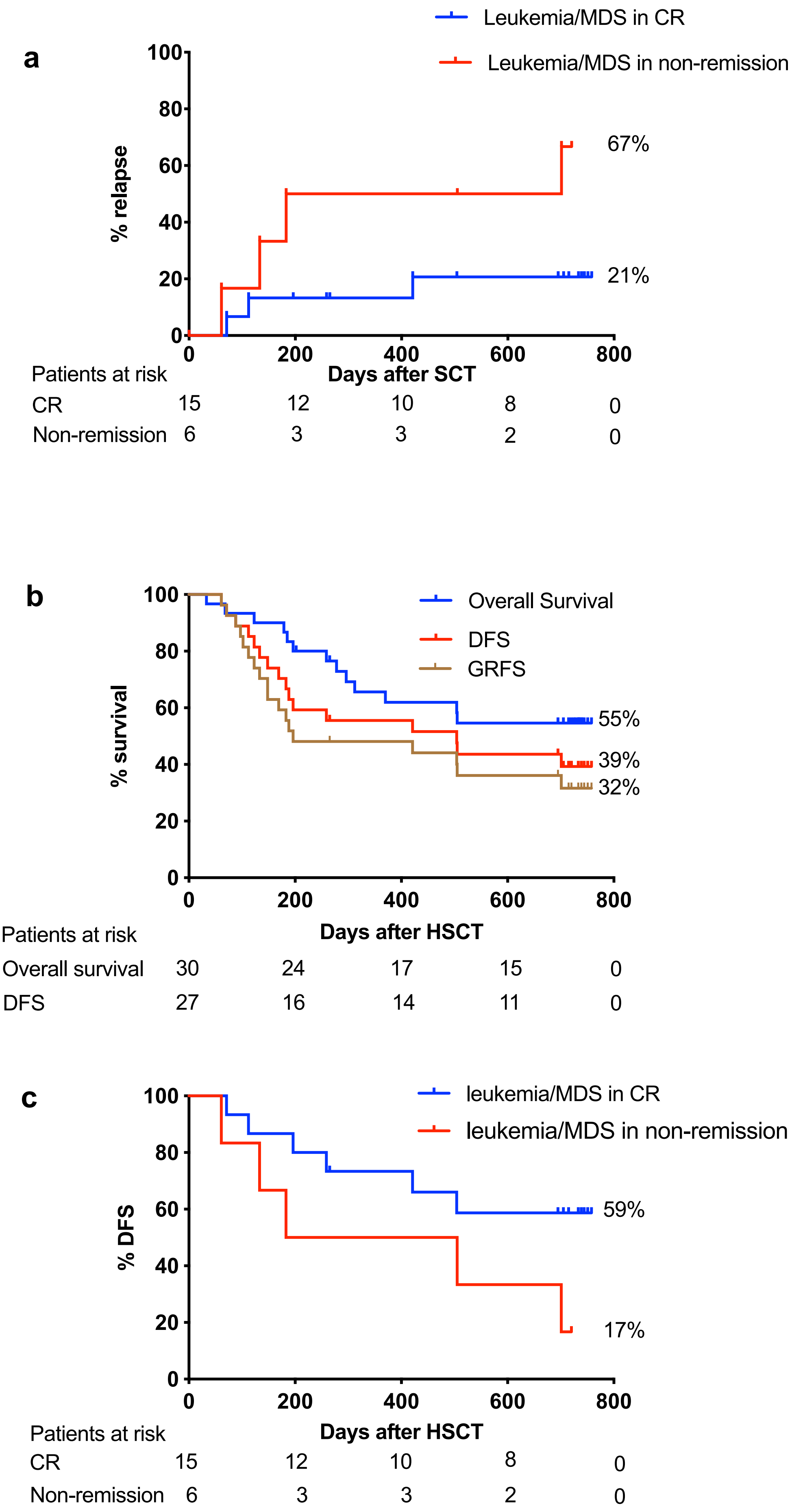
a
Leukemia/MDS in CR
Leukemia/MDS in non-remission
% relapse
67%
21%
Days after SCT
Patients at risk
CR 15 12 10 8 0
Non-remission 6 3 3 2 0
b
Overall Survival
DFS
GRFS
% survival
55%
39%
32%
Days after HSCT
Patients at risk
Overall survival 30 24 17 15 0
DFS 27 16 14 11 0
c
leukemia/MDS in CR
leukemia/MDS in non-remission
% DFS
59%
17%
Days after HSCT
Patients at risk
CR 15 12 10 8 0
Non-remission 6 3 3 2 0

References

1. Déchanet J, Merville P, Lim A, Retière C, Pitard V, Lafarge X *et al.* Implication of gammadelta T cells in the human immune response to cytomegalovirus. *J Clin Invest* 1999; **103**(10)**:** 1437-1449. e-pub ahead of print 1999/05/20; doi: 10.1172/jci5409

2. Austin PC, Lee DS, Fine JP. Introduction to the Analysis of Survival Data in the Presence of Competing Risks. *Circulation* 2016; **133**(6)**:** 601-609. e-pub ahead of print 2016/02/10; doi: 10.1161/circulationaha.115.017719
